# Joint Analysis for Genome-Wide Association Studies in Family-Based Designs

**DOI:** 10.1371/journal.pone.0021957

**Published:** 2011-07-22

**Authors:** Qiuying Sha, Zhaogong Zhang, Shuanglin Zhang

**Affiliations:** 1 Department of Mathematical Sciences, Michigan Technological University, Houghton, Michigan, United States of America; 2 School of Computer Science and Technology, Heilongjiang University, Harbin, China; Centro de Investigación Príncipe Felipe, Spain

## Abstract

In family-based data, association information can be partitioned into the between-family information and the within-family information. Based on this observation, Steen et al. (*Nature Genetics. 2005, 683–691*) proposed an interesting two-stage test for genome-wide association (GWA) studies under family-based designs which performs genomic screening and replication using the same data set. In the first stage, a screening test based on the between-family information is used to select markers. In the second stage, an association test based on the within-family information is used to test association at the selected markers. However, we learn from the results of case-control studies (Skol et al. *Nature Genetics*. 2006, 209–213) that this two-stage approach may be not optimal. In this article, we propose a novel two-stage joint analysis for GWA studies under family-based designs. For this joint analysis, we first propose a new screening test that is based on the between-family information and is robust to population stratification. This new screening test is used in the first stage to select markers. Then, a joint test that combines the between-family information and within-family information is used in the second stage to test association at the selected markers. By extensive simulation studies, we demonstrate that the joint analysis always results in increased power to detect genetic association and is robust to population stratification.

## Introduction

Currently, the family-based association tests such as the TDT and its extensions [1]–[6] are still the most commonly used methods to detect disease susceptibility loci in family-based GWA studies. This kind of methods uses the within-family information, but not the between-family information. The reason is that methods used between-family information may be subject to bias caused by population stratification. Recently, based on the observation that the association information in the family sample can be split into the between-family component and the within-family component, Steen et al. [7] proposed a two-stage test for family-based GWA studies. We call this method Family-based Two-Stage Approach (FTSA). In the first stage of FTSA, a test based on between-family information is used to screen markers, that is, choose *R* “top” markers. In the second stage of FTSA, a family-based association test based on within-family information is used to test the *R* selected markers for association. FTSA is robust to population stratification because the association is determined by the family-based association test in the second stage. Furthermore, since the statistic used in the first stage is statistically independent of that in the second stage, the overall significance level of the algorithm does not need to be adjusted for the first stage. In the following discussion, we call the tests used in the first stage and in the second stage screening test and association test, respectively.

In case-control studies, several authors have proposed a two-stage design which utilizes two independent samples [8], [9]. The first stage that uses the first sample is to screen and select SNPs for association tests. In the second stage, the association tests are conducted on the selected SNPs by using the second sample, so that the number of association tests is diminished and the correction for multiple testing is less severe. Recently, Skol et al. [10] pointed out that joint analysis in which the test used in the second stage is the combination of the two tests based on the two samples is more powerful than replication-based analysis in which the test used in the second stage is based on the second sample only. There are some similarities between FTSA and the two-stage approach in case-control studies. Can we do joint analysis in FTSA as Skol et al. [10] did for the two-stage approach in case-control studies. One problem hindering us from doing joint analysis in FTSA is that the screening test in FTSA can be susceptible to population stratification and thus the joint test in joint analysis that combines the screening test and association test can be also susceptible to population stratification. To overcome this problem, we borrow ideas from methods for case-control studies to construct a screening test that is based on between-family information and also robust to population stratification.

In case-control studies, it has been long recognized that population stratification can seriously confound association results [11], [12]. To overcome this problem, several methods that use a set of unlinked genetic markers, also called genomic markers, genotyped in the same samples have been developed to control for population stratification. These methods can be roughly divided into four groups. The first is genomic control (GC) approach that adjusts the ordinary chi-square test statistic, 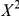. To, 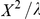 and assumes 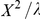 to follow a chi-square distribution, where *λ* can be estimated using genotypes at genomic markers [13]–[15]. The second is “structured association” (SA) that uses a set of independent genetic markers to estimate the number of subpopulations and the ancestry probabilities of individuals from putative “unstructured” subpopulations. This information is then used to test for association [16]–[19]. The third group is principal components (PC) approach that summarizes the genetic background through the PC analysis of genotypes at genomic markers [20]–[24]. The PCs calculated from a matrix of genotypes at genomic markers can be further used to eliminate the effect resulting from population stratification. Zhang et al. [20] and Chen et al. [21] modeled the relationship between trait values, genotype at the candidate marker, and PCs through a semi-parametric model, where the trait value is treated as the dependent variable. Recently, Price et al. [23] presented a linear regression method by regressing both the trait value and genotype at the candidate marker on the PCs. Association between the trait and candidate marker is then tested with the residual correlation. The PC approach is much simpler and computationally faster than the SA approach and is more powerful than the GC approach. The fourth is the mixed linear model (MLM) approach [25], [26] that corrects for a wide range of sample structures by explicitly accounting for pairwise relatedness between individuals.

In this article, we propose a novel approach to do joint analysis within the framework of FTSA. We first perform PC analysis based on parental genotypes at a set of genomic markers and then use a PC approach to eliminate any effect of population stratification both in genotypes at the candidate marker and trait values for all family members. A screening test is then constructed based on adjusted between-family information and parental trait values. We use this screening test which is robust to population stratification to select markers in the first stage. In the second stage, we do joint analysis i.e. use a test that is a combination of the screening test and the association test to test association at the selected markers. The joint analysis is robust to population stratification because both the screening test used at the first stage and the joint test used at the second stage are robust to population stratification. We evaluate the performance of our joint analysis approach by simulation studies under a variety of population admixture models. Our simulation studies show that the proposed joint analysis approach is robust to population stratification and is consistently more powerful than FTSA proposed by Steen et al. [7].

## Methods

Consider a GWA study of 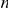 nuclear families with 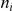. children in the 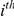. Family 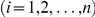 and *L* markers have been genotyped for each sampled individual. For the 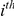. family, we use 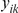. and 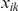. to denote the trait values and genotypic scores at the candidate locus of the, 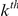. member in the 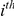. family (*K = 1* and *2* for the two parents), where genotypic score is the number of copies of minor allele.

### Screening Test

We assume that the parental phenotypes are available. In this case, by incorporating parental phenotypes, Feng et al. [27] proposed a screening test statistic
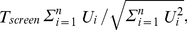
(1)Where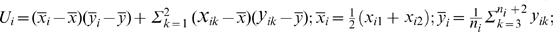 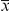 and 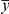 are the overall means of genotypic scores and trait values, respectively. Feng et al. [27] have shown that this test is more powerful than one not incorporating parental phenotypes and is independent of family-based association tests based on within-family information. However, this test may be subject to bias caused by population stratification and thus cannot do joint analysis by combining 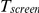. with the test statistic of a family-based association test because the combined test may be also subject to bias caused by population stratification. We previously suggested using the PCs of genotypes at genomic markers to represent the genetic background of unrelated individuals and using the genetic background to control for population stratification in population-based association studies [20]–[22]. We will use this idea to construct a test based on between-family information and incorporating parental phenotypes such that this test is robust to population stratification. To construct the test, we first randomly choose *l* markers from the *L* markers in GWA panel as genomic markers. For the 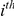 family, let 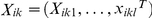 denote multiple marker genotypic scores at the *l* randomly chosen markers of the 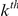. member in the 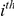. family (*k = 1* and *2* for the two parents). We perform a PC analysis to summarize the genotype data at genomic markers. Because our data are family data, a naive PC analysis with all available data will result in biased directions of maximum variability for the data. Thus, the PC analysis is applied to only the parents in each family.

Let 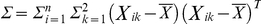 denote the variance-covariance matrix of the genotype data for all of the 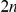 parents, where, 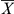. is the overall mean of parental genotypic scores. Let 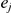 be the 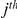 eigenvector corresponding to the 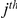 largest eigenvalue of Σ for 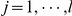. Then, the 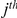. PC for the 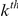 member of the 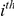 family is given by 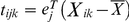 Here we consider only the first *K* PCs (in this study, we use *K = 10*). Because the PCs represent the genetic background information, we adjust both the trait and genotype at candidate loci for this genetic background information by applying linear regression [23]. That is,
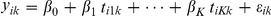
and
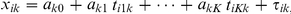
where 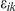 and 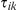 are random errors for 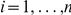 and *k = 1,2*. Let 
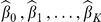
 and 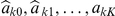 be the least square estimators of 
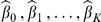
 and, 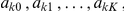 respectively. The residuals of the trait values and genotypic scores at the candidate locus for parents and children of the 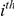. family are calculated by
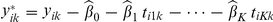
and
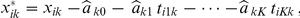
where 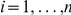 and 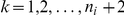. We can consider 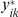 and, 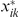 as the trait value and genotypic score of the 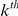. member in the, 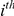. family after adjusted for population stratification.

Based on the adjusted trait values and genotypic scores, we propose the following screening test, called admixture screening (Ascreen) test,
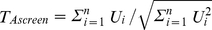
(2)where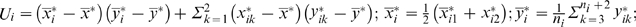 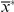 and 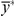.are the overall means of genotypic scores and trait values after adjusted for population stratification, respectively. Under the null hypothesis, 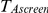 follows a standard normal distribution.

### Association Test

We use quantitative pedigree transmission disequilibrium (QPTD) as our family-based association test [28]. Using the notation given above, the association test statistic is given by
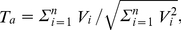
(3)where 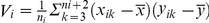 Under the null hypothesis of no association, 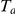. asymptotically follows the standard normal distribution.

### Joint Analysis

In the first stage, we select R markers with the smallest p-values of the admixture screening test. This selection means that there is a constant *C* such that a marker is selected if 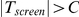. In the joint analysis, a new test statistic
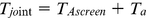
(4)is used to test association in the second stage. Let 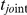. be the observed value of the statistic 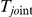., Then, in the second stage, the p-value of the test 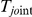. is given by
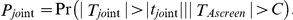
Let 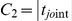 and *T* denote the event of 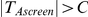. Similarly to equation (2) in Skol et al. [10], we have
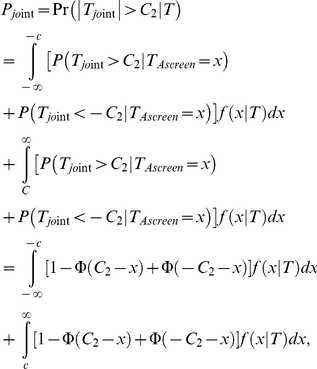
where Φ is the cumulative distribution function of the standard normal distribution; 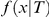 is the probability density function of 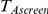 given that 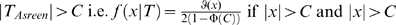 otherwise; 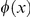 is the probability density function of the standard normal distribution. Thus, the p-value 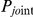 can be calculated numerically. In summary, for the joint analysis, we first select R top markers using the admixture screening test 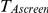 and then test association for each of the R selected markers using the joint test 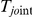. For one of the R selected markers, we declare that this marker is significant at level α if the p-value of the joint test 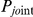 is less than 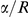.

### Methods Compared

We compare the proposed joint analysis method with two other methods that is described below. One is FTSA proposed by Steen et al [7]. In FTSA, the screening test does not adjust for population stratification. In this study, we use 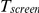 given in equation (1) as the screening test of FTSA. In the first stage of FTSA, R markers with the smallest p-values of the screening test 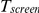 are selected. In the second stage, the association test 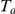 given by equation (4) is used to test each of the R selected markers. A marker in the R selected markers is declared significant at level α if the p-value of the association test 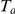 is less than 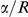. The other method we compare with is a method called Admixture Family-based Two-stage Approach (AFTSA) that is similar to FTSA but replaces the screening test 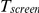 in FTSA by the admixture screening test 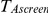 given by equation (2).

## Results

We used simulation studies to compare the performance of the joint analysis with FTSA and AFTSA. We also compared FTST with AFTSA to see if adjusting population stratification in the screening test can improve power of FTSA. The simulation setup used in this study was similar to that of Zhu et al [29]. We considered three sets of simulations: a homogeneous population, a structured population which contained two subpopulations, and an admixture population that mimicked African American population.

### Set 1: A Homogeneous Population

In this set of simulations, we simulated samples based on the haplotype data of 120 European chromosomes (CEU) released by the HapMap project [30]. However, we used only the haplotype data on chromosome 1 at tagging SNPs. There are 34720 SNPs in total. To generate the genotype of a parent, we generated two haplotypes that are the recombinants of the 120 HapMap chromosomes. To generate a recombinant of the 120 chromosomes, we first generated a number of crossovers across the chromosome by a Poisson process with an average of 6 crossovers per Morgan. The crossover locations were generated according to a uniform distribution. The crossover locations divided the chromosome into segments. Each segment of the recombinant was a random chosen haplotype from the HapMap chromosomes in the same segment. The offspring genotypes were generated by randomly transmitting one of the two haplotypes of the father and the mother with the crossovers occurring according to the genetic map. The LD pattern across a chromosome was generally preserved for the SNPs that are closely located.

To generate trait values under the null hypothesis, for a nuclear family with m children, let 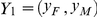 and 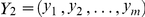 denote the trait values of the parents and the m children. Assumed that 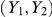 followed a normal distribution with a mean vector of zero and variance-covariance matrix of
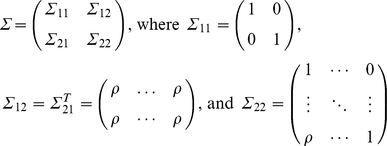



This covariance structure meant that the father and mother were independent, and parents with children and children with children were correlated with the correlation coefficient of *ρ* (in this study, we use *ρ* = 0.4). The conditional distribution of 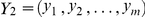 given parental trait values, 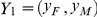 was a normal distribution with mean vector of 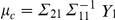. and variance-covariance matrix of 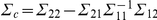. To generate trait values of all individuals in the family, we first generated the trait value of each parent by using a standard normal distribution. The trait values of the children can be generated by a normal distribution with a mean vector of 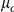 and variance-covariance matrix of 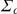, given the trait values of their parents.

Under the alternative hypothesis, we generated the trait values of a nuclear family with *B* members from model 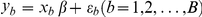 where 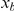. was the numerical code of genotype *g* at the disease locus and
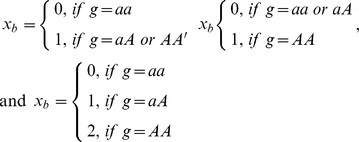



for a dominant, recessive, and additive model, respectively (*a and A* were the two alleles at the disease locus and *A* was the high risk allele); *β* was a constant and 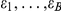 were background trait values generated under the null hypothesis using aforementioned method. The value of *β* was determined by heritability *h* and was given by 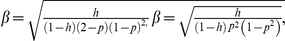, and 
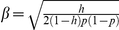
 for a dominant, recessive, and additive model, respectively, where *p* was the allele frequency of the high risk allele.

### Set 2: A Structured Population with Two Subpopulations

In this set of simulations, we simulated samples using the haplotype data of 120 European chromosomes (CEU) and 120 African chromosomes (YRI) released by the HapMap project [30]. In these simulations, we again used only the haplotype data on chromosome 1 at the 34720 tagging SNPs. We considered that all members of a family were from a same subpopulation. The genotypes can be generated in the same way as that in the simulation set 1. In this set of simulations, we sampled 70% of families from European subpopulation and 30% of families from African subpopulation. We generated the trait values of a nuclear family with *B* members from model
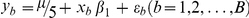



if this family was from European subpopulation and
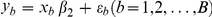
if this family was from African subpopulation, where 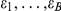 were background trait values generated under the null hypothesis in simulation set 1; 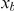 was the numerical code of genotype at the disease locus; *μ* was a constant that measured the difference of the average trait values between the two subpopulations; 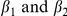 can be determined by heritability *h* and the relationship was given in simulation set 1. We used the same value of *h* in the two subpopulations and thus 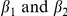 may be different due to the difference of allele frequencies of the high risk allele in the two subpopulations. Furthermore, we set *h* = 0 and thus 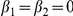 for generating the trait values under the null hypothesis, and *h*>*0* for generating the trait values under the alternative hypothesis.

### Set 3: An Admixture Population with Two Ancestral Populations

Again, we simulated samples based on the chromosome 1 data of 120 European chromosomes and 120 African chromosomes released by the HapMap project [30]. We first generated haplotype exchange points on the chromosome among the populations by using a Poisson process, with an average of 6 crossovers per Morgan. This is equivalent to a population that has been admixed for an average of 6 generations. In each region between two exchange points, we determined which ancestral population a haplotype came from based on a distribution of admixture proportions of Africans and Europeans, which we set to (0.8, 0.2). We then applied the same method as for simulation set 1 to generate a person's genotypes from the selected ancestral population. The method in simulation set 1 for generating offspring genotypes was also applied.

We generated the trait values of a nuclear family with 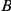 members from model 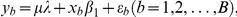 where 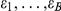 and 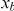 were the same as in simulation set 2; 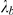 was European admixture proportion of the 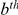 member in the family; *μ* and *β* were constants and *β* can be determined by heritability *h*. Again, we set *h = 0* for generating the trait values under the null hypothesis, and *h*>0 for generating the trait values under the alternative hypothesis.

In all of the three sets of simulations, we used 1000 replicated samples to evaluate the type I error rates and power and considered nuclear family with one child i.e. trio as the family structure. To evaluate the type I error, we considered different sample sizes, different number of markers used to control for population stratification, and different values of *μ*. However, we fixed the value of R, the number of markers selected at the first stage, as 10. We evaluated type I error rates of the three methods (joint analysis, FSTA, and AFSTA) as well as two screening tests 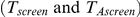. For evaluating type I error rates of the joint analysis, FSTA, and AFSTA, we used 1,000 replicated samples and thus the standard deviation for the type I error rates was 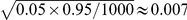 and the 95% confidence interval was (0.036, 0.064) for the nominal level of 0.05. For evaluating type I error rates of 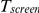 and 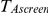 although we still used 1,000 replicated samples, we performed 34720 tests for each sample (equivalent to 16200 independent tests calculated using the method of Gao et al. [31]) and thus the standard deviation for type I error rates was 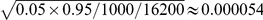 and the 95% confidence interval was (4.989%, 5.011%) for the nominal level of 5%.

Table 1, 2, 3 gave type I error rates of the five tests for simulation set 1 to set 3, respectively. From the three tables, we can see that type I error rates of the joint analysis, FSTA, and AFSTA had the same pattern across simulation set 1 to set 3, i.e. the three tests were slightly conservative. This conservative was probably due to the fact that we used Bonferroni correction to adjust for multiple testing in the second stage. Table 1, 2, 3 showed that although 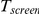 was a valid test in a homogeneous population (Table 1), it would lead to false positive in structured populations (Table 2, 3). Table 1, 2, 3 also showed that 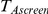 was a valid test in a homogeneous population (Table 1) and it was also a valid test in structured populations if 800 or more genomic markers were used to control for population stratification (Table 2, 3). The non-inflated type I error rates of AFSTA also show that the admixture screening test 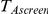 used in the first stage and 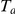 used in the second stage are independent. If 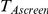 And 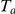 are correlated (either positive or negative correlated), a marker with a small p-value of 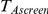 will have a high probability to have a small p-value of 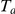, and thus, AFSTA will have inflated type I error rates.

**Table 1:** Type I error rates (in percentage) of the five tests based on simulation set 1.

		Method				
S	L	Joint	FTSA	AFTSA	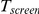	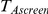
400	200	3.3	4	3.7	4.99713	5.0091
	400	3.8	2.8	3.1	4.99812	5.00628
	800	2.7	3.5	3.6	4.98895	5.01246
600	200	3.5	3	3.4	4.99385	5.00721
	400	3.3	3.3	3.7	4.98347	5.00162
	800	3.2	3.7	3.8	4.98031	5.01397
800	200	3.5	4.1	5.3	4.99007	4.99439
	400	4.4	4.4	5.0	4.99462	5.01143
	800	2.9	3.8	3.8	4.99412	5.01168

Significant level is 5%.

Note: S denotes sample size in trios; L denotes the number of genomic markers used to control population stratification.

**Table 2:** Type I error rates (in percentage) of the five tests based on simulation set 2.

		*μ* = 1					*μ* = 2				
S	L	Joint	FTSA	AFTSA	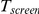	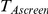	Joint	FTSA	AFTSA	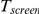	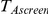 .
400	200	3.7	3.3	4.2	69.9	5.41605	4.3	4.5	4.3	80.3	6.53089
	400	3.4	4.2	4.9	69.9	5.15228	3.5	3.9	4.2	80.4	5.4674
	800	4.1	2.8	4.6	69.9	5.00927	3.3	3.1	4.5	80.3	5.0182
600	200	3.6	2.9	3.3	75.3	5.5889	4.0	3.2	4.4	83.9	7.29293
	400	3.9	3.8	3.1	75.3	5.18648	4.1	3.6	4.0	83.9	5.6435
	800	3.4	4.4	4.5	75.3	5.00261	4.2	4.1	4.9	83.9	5.01144
800	200	3.5	3.9	3.6	78.5	5.77735	3.7	3.9	3.1	85.9	7.99526
	400	4.5	4.6	4.6	78.4	5.20667	4.8	5.2	5.7	85.9	5.81495
	800	3.7	3.2	3.8	78.4	5.01154	3.4	4.1	3.1	85.9	5.01228

Significant level is 5%.

Note: S denotes sample size in trios; L denotes the number of genomic markers used to control population stratification.

**Table 3:** Type I error rates (in percentage) of the five tests based on simulation set 3.

		*μ* = 1					*μ* = 2				
S	L	Joint	FTSA	AFTSA	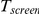	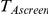	Joint	FTSA	AFTSA	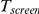	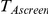
400	200	4.1	2.9	3.3	5.5	5.31204	4.2	3.6	3.6	7.0	6.06805
	400	3.8	4	4.5	5.5	5.17645	4.2	3.8	3.8	7.0	5.56551
	800	3.4	3.8	3.3	5.5	5.01086	4.2	3.8	3.5	7.0	5.01277
600	200	4.4	3.2	3.8	5.8	5.34633	6.5	4.7	4.7	8.1	6.37518
	400	4.7	4.1	2.9	5.8	5.23698	6.5	5.3	2.9	8.1	5.75471
	800	2.9	4.1	2.8	5.8	5.00985	4.1	7.1	3.5	8.1	5.01071
800	200	3.5	3.8	4.2	6.0	5.48167	5.9	5	5.4	9.1	6.85698
	400	3.7	4.2	3.4	5.7	5.30941	5.0	6.6	4.4	8.7	6.01961
	800	3.3	3.6	3	6.1	5.01124	5	5.1	4.4	9.1	5.01138

Significant level is 5%.

Note: S denotes sample size in trios; L denotes the number of genomic markers used to control population stratification.

**Figure 1:**
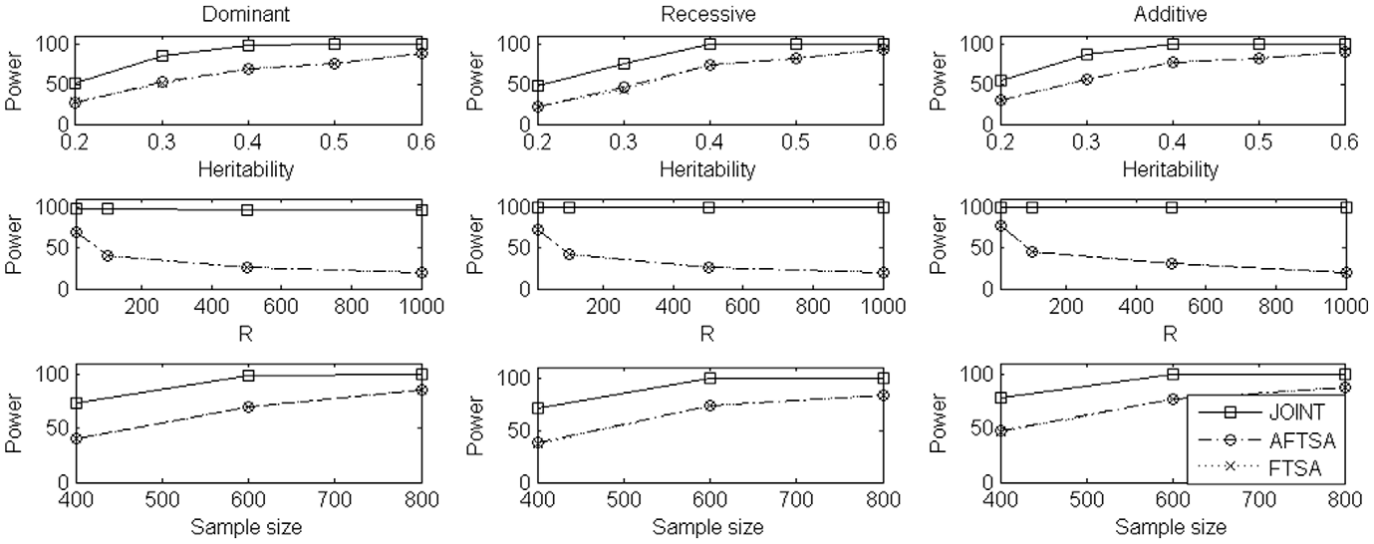
Power comparison under simulation set 1 when *μ* = 2. In the first row, we compare power of the three methods for different values of heritability under the three disease models while sample size is 600 trios and the number of markers selected at the first stage is 10. In the second row, we compare power of the three methods for different numbers of markers selected at the first stage under the three disease models while sample size is 600 trios and heritability is 0.05. In the third row, we compare power of the three methods for different sample sizes under the three disease models while heritability is 0.05 and the number of markers selected at the first stage is 10. In each case, we use 800 genomic markers to control for population stratification in the admixture screening test.

**Figure 2:**
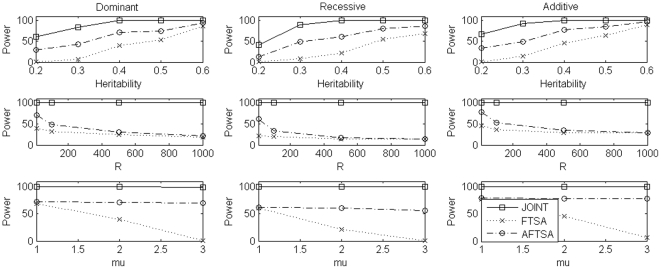
Power comparison under simulation set 2 when sample size is 600 trios. In the first row, we compare power of the three methods for different values of heritability under the three disease models while *μ* = 2 and the number of markers selected at the first stage is 10. In the second row, we compare power of the three methods for different numbers of markers selected at the first stage under the three disease models while *μ* = 2 and heritability is 0.05. In the third row, we compare power of the three methods for different values of *μ* under the three disease models while heritability is 0.05 and the number of markers selected at the first stage is 10. In each case, we use 800 genomic markers to control for population stratification in the admixture screening test.

**Figure 3:**
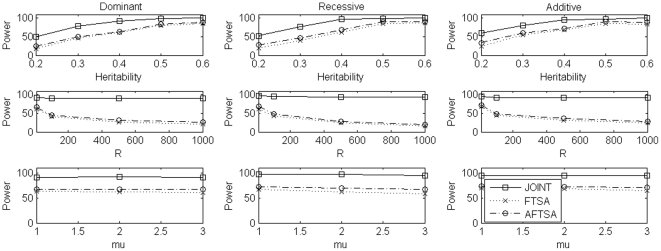
Power comparison under simulation set 3 when sample size is 600 trios. In the first row, we compare power of the three methods for different values of heritability under the three disease models while *μ = 2* and the number of markers selected at the first stage is 10. In the second row, we compare power of the three methods for different numbers of markers selected at the first stage under the three disease models while *μ = 2* and heritability is 0.05. In the third row, we compare power of the three methods for different values of *μ* under the three disease models while heritability is 0.05 and the number of markers selected at the first stage is 10. In each case, we use 800 genomic markers to control for population stratification in the admixture screening test.

For power comparison, we considered different scenarios which included different values of heritability *h*, different number of markers selected at the first stage, different values of *μ*, and different sample sizes. To evaluate power, in each replication, we randomly chosen a marker with minor allele frequency (calculated from European subpopulation in simulation set 2) in the interval (0.1, 0.3) as the disease locus and the minor allele as the high risk allele for dominant and additive models while the major allele as the high risk allele for recessive model. Results of power comparison were summarized in Figure 1, 2, 3 for simulation set 1 to set 3, respectively. Under simulation set 1, which considered a homogeneous population, the joint analysis was consistently more powerful than FSTA and AFSTA. Also, FSTA and AFSTA had almost the same power (Figure 1). These results indicate that the admixture screening test, robust to population stratification, did not lose power when compared to the traditional screening test. In simulation set 2, we considered a structured population with two subpopulations. In this set of simulations, the joint analysis was again consistently more powerful than the other two methods, and AFSTA was consistently more powerful than FSTA, which showed that using the admixture screening test instead of traditional screening test increased power in the presence of population stratification (Figure 2). In simulation set 3, we considered an admixture population with two ancestral populations which also leaded to the problem of population stratification but not as strong as that in simulation set 2. In this set of simulations, the pattern of power comparison was very similar to that in simulation set 2, but the power difference between FSTA and AFSTA was not as large as that in simulation set 2 (Figure 3). In summary, the joint analysis was consistently the most powerful one among the three methods we considered. Comparing the other two methods, AFSTA had almost identical power with FSTA in the case of no population stratification and was more powerful than FSTA in the presence of population stratification.

## Discussion

In this article, we proposed a novel method to perform joint analysis within the framework of the family-based two-stage analysis. In the joint analysis, we first constructed a screening test that was based on between-family information and was robust to population stratification. In the first stage, we used this screening test to select markers. In the second stage, we did joint analysis i.e. used a test that was a combination of the screening test and the association test to test association at the selected markers. The joint analysis was robust to population stratification because both the screening test and the association test are robust to population stratification. Our simulation studies showed that the joint analysis was consistently more powerful than two-stage approaches in which the association test used in the second stage was only based on within-family information.

Although we have discussed the joint analysis, in which we only tested the selected markers in the second stage, it is straightforward to extend the joint analysis to the p-value weighting scheme [32], [33] in which, instead of testing selected markers only, all markers are tested in the second stage and the resulting p-values are weighted using the p-values of the screening test. Using the p-value weighting scheme, the following steps can be used to perform the joint analysis. (1) Test all SNPs using the admixture screening test 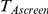 and order SNPs according to their p-values of the test. (2) Divide the SNPs into groups with the first group containing 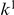 SNPs and the 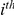 group containing 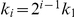 SNPs. (3) Let 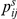 denote the p-value of the admixture screening test at the 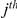 SNP in the 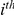 group and 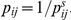 Define an importance measure 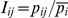 and a weight 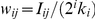 for the 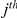 SNP in the 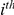 group, where 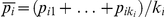 (4) Test each SNP using the joint test statistic 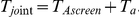 Denote 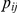 the p-value of the joint test at the 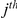 SNP in the 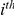 group. Then, declare the 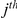 SNP in the 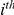 group to be significant at a level of α if 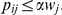 For the method of Ionita-Laza et al [32], 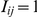 Furthermore, for simplicity, we discussed our method using nuclear families. Our method can be also applied to general pedigrees. In fact, both the screening test 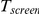 given by equation (1) and association test 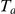 given by equation (3) are applicable to general pedigrees [27], [28].

It should be noted that the PC approach used in 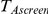 to control for population stratification may be not as strongly resistant to stratification bias as the approach in Steen et al. [7] in which the significant association totally depends on the family-based association test used in the second stage. Other problems of the PC approach include (1) there is no standard as to how many PCs should be used; (2) the PC approach uses additive coding to code the population structure and also assumes additivity between the effects of the PCs and the effects of the genomic markers. According to our experience of using the PC approach, however, if we use all markers in a GWAS as genomic markers, the first 10 PCs can capture subtle population structures such as the population structure in European Americans.

One remaining question is choosing the value of *R*, the number of markers selected in the first stage. Although there is no unique answer in choosing an optimal value of *R*, our simulations indicate that 10 is a good choice of *R* which is consistent with the results of Steen et al [7]. However, we need further investigations on choosing the optimal value of *R* in general.
